# Mixed Plasmodium vivax and falciparum Malaria in a Young Infant: The Critical Role of Peripheral Smears in an Endemic Region

**DOI:** 10.7759/cureus.113330

**Published:** 2026-07-24

**Authors:** Aleena Rizvi, Mahima Choudhary, Shikha Agarwal, Divya Sachdeva, Utkarsh Bansal

**Affiliations:** 1 Pathology, Hind Institute of Medical Sciences, Barabanki, IND; 2 Pediatrics, Hind Institute of Medical Sciences, Barabanki, IND

**Keywords:** artemisinin-based combination therapy (act), chloroquine, dual plasmodium infection, mixed-species malaria, pediatric malaria, plasmodium co-infections, primaquine radical cure, rapid diagnostic test, thick smear, thin smear

## Abstract

Malaria remains a major global public health challenge, particularly in endemic regions and among travelers. Mixed-species malarial co-infections in these areas are frequently underdiagnosed or underestimated due to their lower incidence, especially in vulnerable pediatric populations. Such co-infections often result in severe complications and delayed targeted therapy. Here, we report the case of an 18-month-old male infant who presented with a low-grade fever, abdominal pain, severe anemia, and hepatosplenomegaly. Given the regional endemicity and season, *Plasmodium vivax* infection was initially suspected, and empirical treatment was initiated. However, a peripheral blood smear subsequently revealed a mixed infection with *Plasmodium falciparum*, necessitating an immediate adjustment in the therapeutic regimen. Following the targeted intervention, the patient demonstrated significant clinical and hematological recovery. This case emphasizes the critical importance of considering mixed plasmodial co-infections in pediatric patients presenting with non-specific symptoms and rapid clinical deterioration in endemic areas. Microscopic examination of peripheral smears by a trained pathologist remains the gold standard for early identification, which is essential to secure favorable outcomes in complex pediatric cases.

## Introduction

Malaria is an acute, potentially life-threatening parasitic disease caused by protozoa of the genus *Plasmodium* and transmitted to humans via the bite of infected female *Anopheles* mosquitoes [1]. Five distinct species are known to cause malaria in humans: *Plasmodium falciparum*, *Plasmodium vivax*, *Plasmodium ovale*, *Plasmodium malariae*, and *Plasmodium knowlesi*. Among these, *P. vivax* is the most widely distributed geographically, whereas *P. falciparum* is responsible for the highest morbidity and mortality rates [2].

Globally, an estimated two billion people reside in malaria-endemic zones across approximately 90 countries, with an additional 125 million travelers at risk annually [1]. According to recent World Malaria Reports, global cases have hovered around 260 million annually, resulting in nearly 600,000 deaths, with approximately 95% of this mortality burden concentrated in the WHO African Region [2]. India accounts for roughly 2% of the global malaria burden, with *P. vivax* and *P. falciparum* representing the predominant species nationwide [3].

Individuals residing in or traveling to endemic areas can contract mono or mixed infections with *Plasmodium* species. In pediatric populations, mixed infections are rare. They are most frequently observed in children aged two to five years and are notably less common in infants under two years of age [4]. Crucially, children under five years remain the most vulnerable demographic, accounting for approximately 73% of all malaria-related deaths worldwide [2]. Here, we report a rare case of a mixed *P. vivax* and *P. falciparum* infection in an 18-month-old male infant who initially presented with low-grade fever and abdominal pain.

## Case presentation

An 18-month-old male infant presented to the pediatric outpatient department at the Hind Institute of Medical Sciences (Safedabad, Barabanki, Uttar Pradesh, India) in September 2025 with a 15-day history of fever and abdominal pain. The past medical and family histories were unremarkable. The fever was low-grade, intermittent, undocumented at home, and temporarily responsive to antipyretics. The abdominal pain was severe, manifested by inconsolable crying and a refusal to feed.

Upon physical examination, the infant appeared irritable and severely pale, with icteric mucous membranes. Vital signs revealed a pulse rate of 148 beats per minute, a respiratory rate of 37 breaths per minute, and a temperature of 102°F. Abdominal examination demonstrated tender hepatomegaly (extending 3 cm below the right subcostal margin) and splenomegaly (extending 3 cm below the left subcostal margin). The infant was predominantly breastfed. Given the seasonal transmission patterns and regional endemicity, *P. vivax *malaria complicated by congestive heart failure was provisionally suspected, and empirical management was initiated. Blood samples were drawn for a rapid diagnostic test (RDT) for malaria, serology of common tropical infections, as well as complete hematological and biochemical profiles.

**Figure 1:**
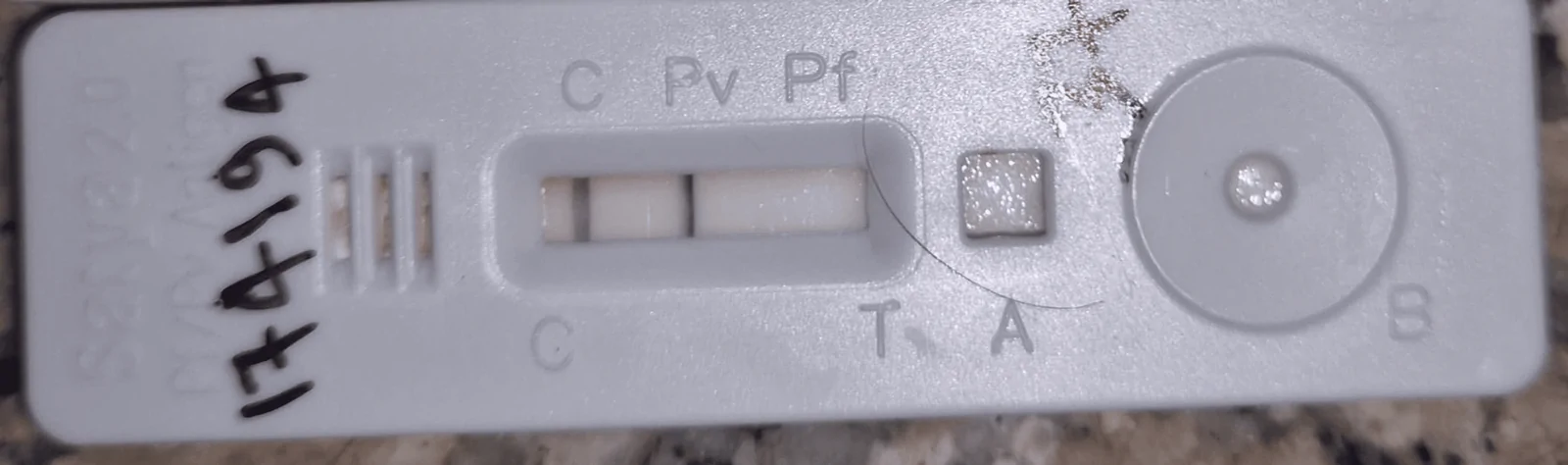
First Rapid Diagnostic Test (RDT) Cassette Result Distinct immunochromatographic bands are visible at the control line (C) and the *Plasmodium vivax*-specific line (Pv), confirming a *Plasmodium vivax *infection. Satya 2.0 Pf/Pv Malaria Antigen Card (Viola Diagnostic Systems, Rudrapur, India).

The initial RDT was positive exclusively for *P. vivax* (Figure 1).

**Figure 2:**
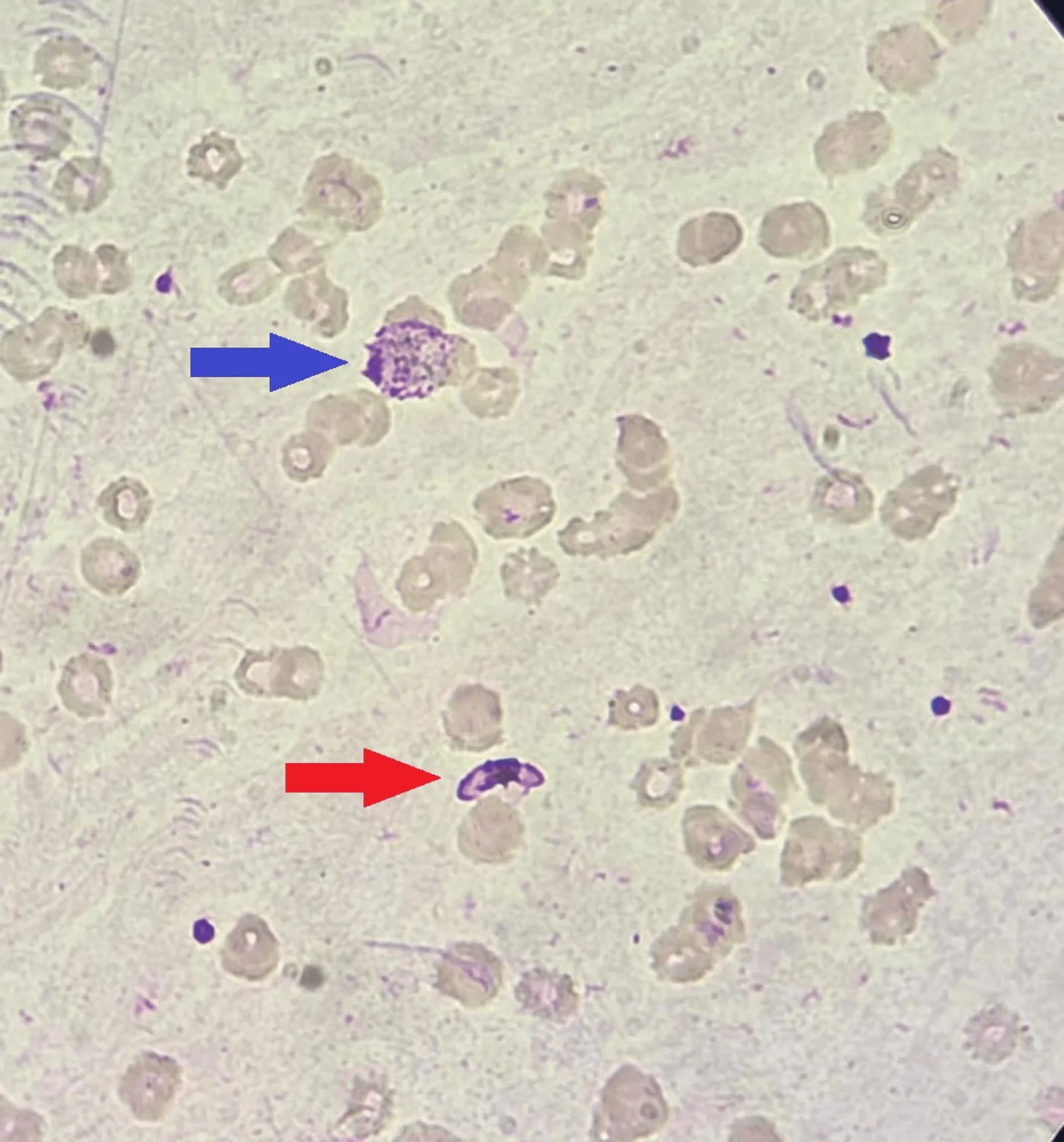
Peripheral Blood Smear on the Day of Admission Gametocytes of both *Plasmodium vivax* (blue arrow) and *Plasmodium falciparum* (red arrow) visible. Magnification: 100x (oil immersion), stain: Romanowsky stain (Leishman stain).

However, a thick peripheral blood smear examined by the pathologist demonstrated the concurrent presence of both* P. falciparum* and *P. vivax* gametocytes. Gametocytes of* P. vivax* are round or oval in shape, containing dispersed brown pigments that are compact and Schüffner's dots, while those of *P. falciparum* are curved, banana- or crescent-shaped with pointed ends, deep blue cytoplasm and tightly packed chromatin in the center (Figure 2).

**Figure 3:**
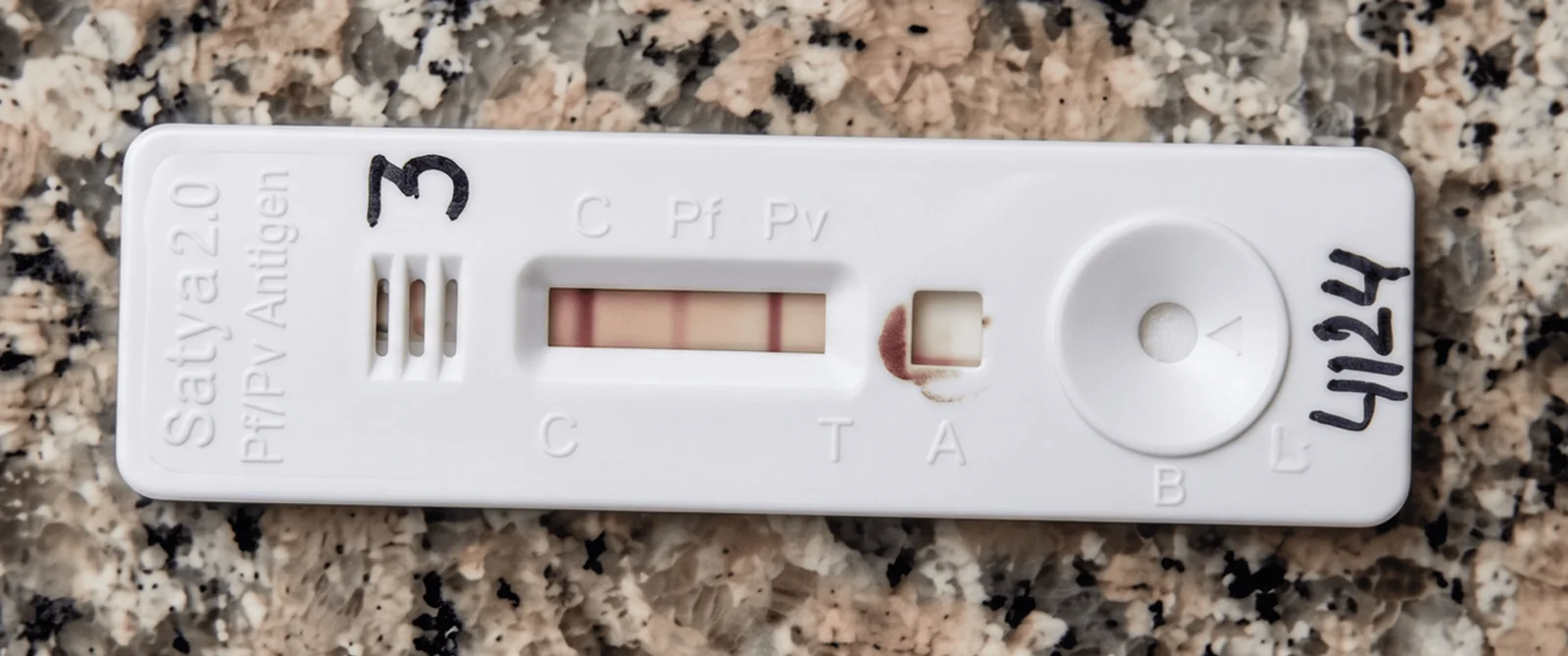
Second Rapid Diagnostic Test (RDT) Cassette Result Distinct immunochromatographic bands are visible at the control line (C), the *Plasmodium falciparum*-specific line (Pf), and the *Plasmodium vivax*-specific line (Pv), confirming a dual parasitic burden. Satya 2.0 Pf/Pv Malaria Antigen Card (Viola Diagnostic Systems, Rudrapur, India).

Following the microscopic findings, a repeat RDT was performed, which confirmed positivity for both *P. falciparum* and *P. vivax*, thereby corroborating the smear diagnosis (Figure 3). The RDT used initially consisted of Pf antigen that detects *P. falciparum*-specific histidine-rich protein-2 (Pf HRP-2) and Pv antigen that detects *P. vivax*-specific Plasmodium lactate dehydrogenase (pLDH). Initially, the specific strain RDT could not detect the Pf antigen, which may be due to its very low levels in the blood. As the infection progresses, the population of parasites in the blood changes. When the strain that does produce the HRP2 protein multiplies and becomes dominant, it floods the bloodstream with the protein. When this happens, the RDT can finally detect the HRP2 protein and will show a true positive.

The complete blood count revealed severe anemia without initial thrombocytopenia (Table 1).

**Table 1:** Longitudinal Hematological Profiles Across the Treatment Course WBC: White Blood Cell, PCV: Packed Cell Volume, MCV: Mean Corpuscular Volume, MCH: Mean Corpuscular Hemoglobin, MCHC: Mean Corpuscular Hemoglobin Concentration, RBC: Red Blood Cell, RDW-CV: Red Cell Distribution Width-Coefficient of Variation, *P. falciparum*: *Plasmodium falciparum*,* P. vivax*: *Plasmodium vivax*

Test Parameter	Day 1	Day 4	Day 8	Day 15	Reference Range
Hemoglobin (g/L)	59	106	111	125	105-135
WBC (× 10^9^/L)	4.20	5.40	7.60	8.20	6.00-17.00
Neutrophils (%)	28	34	40	40	15-46
Lymphocytes (%)	70	63	55	56	45-76
Monocytes (%)	2	3	4	3	3-6
Eosinophils (%)	0	0	1	1	0-3
Basophils (%)	0	0	0	0	0-2
PCV (%)	16.6	30.7	32.4	39.5	33-49
MCV (fL)	66.7	83.0	85.3	85.1	70-86
MCH (pg)	23.6	28.7	29.3	29.2	23-31
MCHC (g/L)	354	345	343	341	300-360
Platelet count (× 10^9^/L)	160	100	150	220	150-450
RBC count (× 10^12^/L)	2.48	3.70	3.80	4.00	3.70-5.30
RDW-CV (%)	17.1	31.3	29.3	16	11.5-16.0
Reticulocyte Count (%)	0.8	0.7	-	4.3	0.5-2.0
Peripheral blood smear	Gametocytes of P. falciparum and P. vivax were detected	Gametocytes of P. falciparum were detected	No malaria parasite detected	No malaria parasite detected	-

Biochemical analysis showed elevated direct bilirubin levels, indicating active hemolysis (Table 2).

**Table 2:** Longitudinal Biochemical Profiles Across the Treatment Course CRP: C-Reactive Protein, SGPT: Serum Glutamic Pyruvic Transaminase, SGOT: Serum Glutamic Oxaloacetic Transaminase

Test Parameter	Day 1	Day 4	Day 15	Reference Range
CRP Titre (mg/L)	73.9	4.2	3.2	0-5
Liver Function Tests				
Bilirubin Total (mg/dL)	2.0	1.2	0.8	0.3-1.3
Bilirubin Direct (mg/dL)	1.0	0.30	0.2	≤ 0.25
SGPT (IU/L)	9.5	10.0	18	5-45
SGOT (IU/L)	17.0	15.0	22.5	5-35
Alkaline Phosphatase (IU/L)	230.5	200.0	170.0	30-310
Kidney Function Tests				
Urea (mg/dL)	18.41	-	20.86	16.8-43.2
Creatinine (mg/dL)	0.26	-	0.52	0.8-1.3 (Male), 0.5-0.9 (Female)
Sodium (mmol/L)	134.7	-	138.1	135-145
Potassium (mmol/L)	4.74	-	4.2	3.5-5.2
Calcium (mg/dL)	9.13	-	9.82	8.5-11.0
Albumin (g/dL)	3.5	-	4.2	3.6-5.1
Total protein (g/dL)	6.0	-	6.5	6.6-8.3

Diagnostic evaluations for concurrent endemic illnesses, including serology for dengue, typhoid, leptospirosis, and scrub typhus, were negative. Urine routine and microscopic evaluation were normal. The urine and blood cultures were sterile.

Consequently, the working diagnosis was revised to a mixed-species malaria infection, necessitating an immediate modification of the therapeutic regimen. The patient had initially been started on oral chloroquine, but following the smear confirmation, the treatment was shifted to intravenous artesunate. The patient’s glucose-6-phosphate dehydrogenase (G6PD) levels were confirmed within normal limits before the initiation of radical therapy with a 14-day course of primaquine. In the management of severe anemia and secondary congestive heart failure, the patient was transfused with two units of packed red blood cells. He was monitored daily, showing progressive clinical and hematological stabilization. 

**Figure 4:**
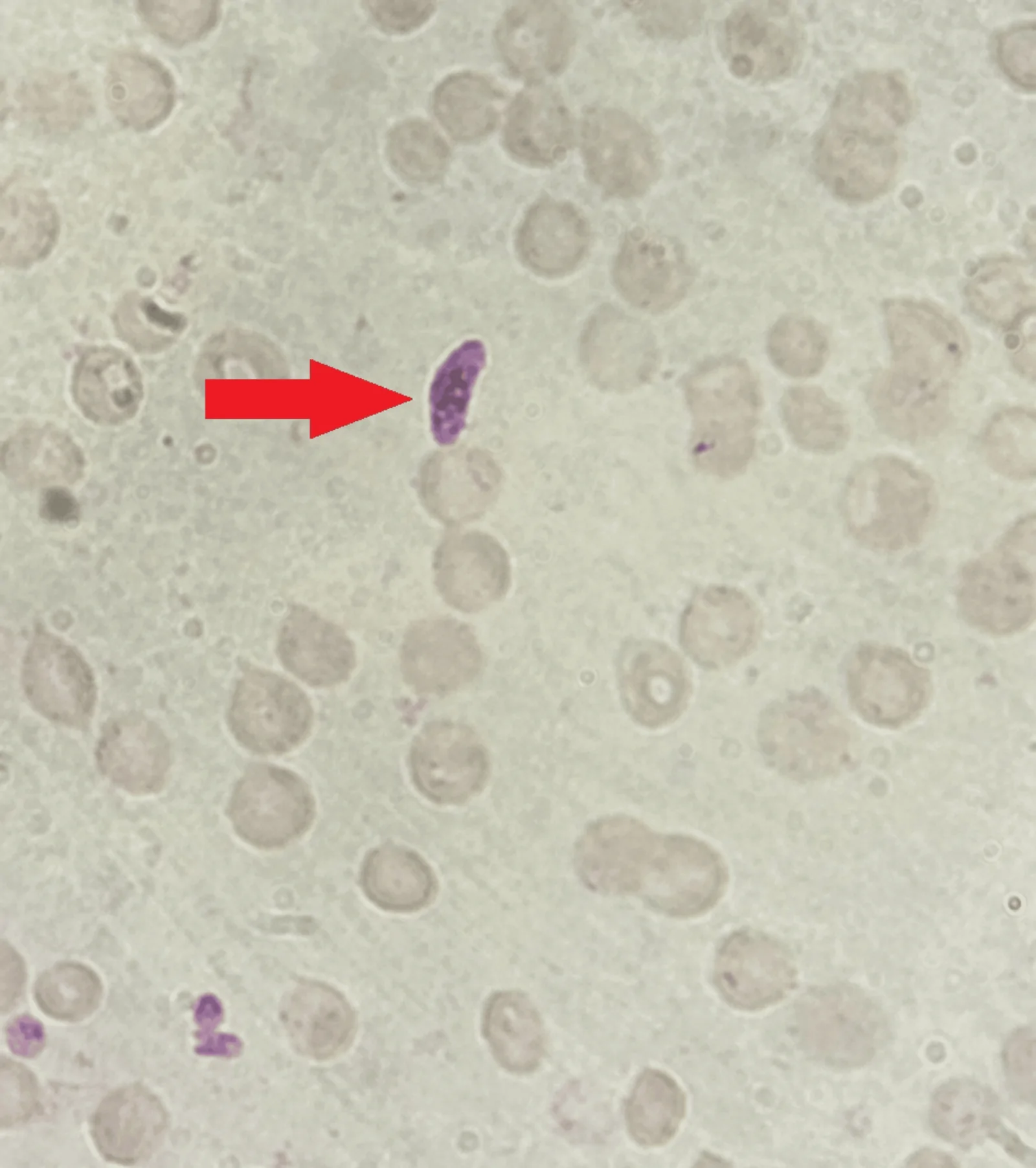
Peripheral Blood Smear on Day Four of Admission Gametocytes of *Plasmodium falciparum* (red arrow). Magnification: 100x (oil immersion), stain: Romanowsky stain (Leishman stain).

In the subsequent course, on day four, the patient demonstrated a minor drop in platelet counts and the presence of only *P. falciparum* gametocytes (Table 1, Figure 4).

On day eight, all counts were within the normal range, and no malarial parasite was detected on the peripheral blood smear (Table 1, 2). The patient gradually improved with no complications and was stable at the time of discharge. The family was given counselling for complementary feeding and mosquito control measures. On follow-up, the patient was completely well. There was a good appetite, and he was accepting complementary feeds well. The vitals were normal and abdominal examination demonstrated regression of hepatomegaly and splenomegaly (extending 1 cm below the right and left subcostal margins respectively).

## Discussion

India remains highly endemic for malaria, with* Plasmodium* monoinfections representing a significant portion of the seasonal disease burden [3]. While mixed-species infections occur in these regions, they are notably less frequent, particularly among infants and young children. The presence of co-infection in pediatric patients can exponentially increase disease severity and the risk of life-threatening complications, including cerebral malaria, severe anemia, and respiratory distress [5]. Other systemic manifestations may include jaundice, acute renal failure, hypoglycemia, and circulatory collapse, which can lead to permanent organ damage or neurodevelopmental delays if not addressed promptly [6].

In the present case, despite the presence of dual species and the development of secondary congestive heart failure, the patient responded favorably to intervention. This successful outcome is likely attributable to early diagnostic detection and the prompt initiation of targeted therapy. Furthermore, the patient’s predominantly breastfed status may have played a protective role; ingestion of maternal antibodies likely conferred passive immunity, potentially modulating the inflammatory response and limiting the progression to severe complications. This observation aligns with findings by Ehouman et al., who reported a mixed infection in a breastfeeding one-year-old female who presented with anemia but remained otherwise stable [7].

The longitudinal hematological and biochemical profiles in this infant provide compelling insights into the kinetics of infection resolution and treatment response. On admission (Day 0), the patient exhibited an intensely elevated C-reactive protein (CRP) level of 73.9 mg/L, reflecting severe systemic inflammation driven by the dual parasitic load [8]. This was accompanied by marked hyperbilirubinemia (total bilirubin: 2.0 mg/dL; direct bilirubin: 1.0 mg/dL), indicating active, severe intravascular hemolysis typical of heavy plasmodial burdens. Following targeted therapy, the dramatic drop in CRP to 4.2 mg/L by Day 8 and the normalization of total bilirubin to 0.8 mg/dL by Day 15 serve as objective biochemical proxies for rapid parasite clearance, reduction of systemic inflammatory stress, and the stabilization of erythrocyte membranes.

A particularly noteworthy hematological phenomenon was observed in the red cell distribution width (RDW-CV) trends. On Day 0, the baseline RDW-CV was slightly elevated at 17.1%, consistent with microcytic anemia. However, following the therapeutic transfusion of two units of packed red blood cells, the RDW-CV increased sharply to 31.3% by Day 4 and remained elevated at 29.3% on Day 8. This profound, temporary spike represents a classic presentation of marked anisocytosis. In this clinical context, it directly reflects the dimorphic erythrocyte population co-existing within the infant's circulation: the patient’s residual microcytic host cells blended with the newly introduced normocytic donor red blood cells. By Day 15, as erythropoiesis stabilized and iron indices equilibrated, the RDW-CV normalized to 16.0%, providing clear evidence of hematological recovery [9].

Mixed malarial infections are exceptionally rare in the infant demographic within the Indian subcontinent, particularly during the high-transmission post-monsoon month of September [10]. National guidelines for such cases recommend prioritizing the management of the* P. falciparum* component due to its higher lethality. While oral chloroquine is sufficient for uncomplicated *P. vivax*, the identification of a mixed infection with severe anemia and secondary congestive heart failure mandates immediate escalation to intravenous artesunate [11]. Nutritional anemia is common in this part of the world, especially in infants receiving inadequate complementary feeding, including both iron-deficiency and vitamin B12-deficiency anemia [12]. Furthermore, to achieve a radical cure and eliminate hepatic hypnozoites, a 14-day course of primaquine is indicated. In this case, confirming that the patient’s G6PD levels were within normal limits prior to administration was an essential step to mitigate the risk of drug-induced acute hemolytic crisis in a baseline-anemic infant [13].

Our findings highlight the clinical superiority and paramount importance of meticulous peripheral smear examination over standalone RDTs. In this instance, the initial RDT failed to detect *P. falciparum*, a pitfall that could have led to inadequate monotherapy and catastrophic clinical decline [14]. The gametocytes of *P. falciparum* and *P. vivax* can be distinguished morphologically in the peripheral smear. The gametocytes of *P. falciparum* appear as distinct, banana- or crescent-shaped cells that contain coarse, dark hemozoin pigment clustered in the center, while those of *P. vivax* appear large, round to oval, nearly fill the swollen host red blood cell, and consist of a compact nucleus, brown pigments, and Schüffner's dots. Unlike *P. falciparum*, whose gametocytes require 10-12 days of deep-tissue sequestration to mature and appear later in infection, *P. vivax* gametocytes mature rapidly and appear in the peripheral blood early alongside asexual stages. Microscopic validation by the pathologist not only corrected the diagnostic trajectory but guided the successful clinical resolution, underscored by the negative screens for co-endemic pathogens like dengue, typhoid, leptospirosis, and scrub typhus [15].

This case underscores the necessity for clinicians and pathologists to maintain a high index of suspicion for mixed infections in pediatric patients presenting with non-specific febrile illness during peak transmission seasons. In this instance, the definitive diagnosis was achieved via microscopic examination of a thick smear, which corrected the initial false-negative result for *P. falciparum* provided by the RDT. Consequently, the role of the pathologist is vital in guiding diagnostic accuracy and subsequent therapeutic shifts. We emphasize that peripheral smears should be routinely prepared for all febrile pediatric patients in endemic zones to ameliorate the risk of missed diagnoses.

## Conclusions

This report details a rare mixed infection of *P. vivax* and *P. falciparum* in an 18-month-old male infant presenting with intermittent fever, severe anemia, and secondary congestive heart failure in September 2025. The case underscores the diagnostic pitfalls of relying solely on baseline RDTs and reaffirms the status of peripheral blood smears as the definitive gold standard. Furthermore, it demonstrates that timely intervention with intravenous artesunate, safe G6PD-guided radical therapy with primaquine, and packed red blood cell transfusion can yield excellent outcomes without residual complications in vulnerable pediatric populations.
